# Full-length chloroplast genome of Dongxiang wild rice reveals small single-copy region switching

**DOI:** 10.3389/fpls.2022.929352

**Published:** 2022-09-29

**Authors:** Jianguang Liang, Rui Chen, Fantao Zhang, Qian Wang, Yingxia Yang, Mingjie Lv, Shuangyong Yan, Shan Gao

**Affiliations:** ^1^ School of Pharmacy, Changzhou University, Changzhou, China; ^2^ Institute of Crop Germplasm and Biotechnology, Tianjin Academy of Agricultural Sciences, Tianjin, China; ^3^ Tianjin Institute of Crop Research, Tianjin Academy of Agricultural Sciences, Tianjin, China; ^4^ College of Life Sciences, Jiangxi Normal University, Nanchang, China; ^5^ College of Life Sciences, Nankai University, Tianjin, China

**Keywords:** structural variation, inverted repeat, asymmetric recombination, SDSA, transposon-like element

## Abstract

**Background:**

Plant chloroplast DNA (cpDNA) typically has a circular structure, including a large single-copy region (LSC), a small single-copy region (SSC) and two inverted repeats (IR1 and IR2). The organization of these four elementary regions LSC-IR1-SSC-IR2 is highly conserved across all plant cpDNAs. Very few structural variations (SVs) occurring at the elementary-region level have been reported.

**Results:**

In the present study, we assembled the full-length cpDNA of Dongxiang wild rice line 159 (DXWR159). Using the long PacBio subreads, we discovered a large inversion of SSC and a large duplication of IR in DXWR159 cpDNAs. Significantly, we reported for the first time forward and reverse SSCs of cpDNAs in similar proportions and named the frequent inversion of a whole SSC as SSC switching.

**Conclusions:**

Our study helps researchers to correctly assemble the chloroplast genomes. Our recombination model explained the formation of large SVs in cpDNAs and provided insights into a novel scientific question that if there are common mechanisms in the formation or translocation of all kinds of transposon-like elements (TLEs). We propose that: (1) large inversion is the most accepted mutation type of SVs in cpDNAs; (2) SSC switching ubiquitous occurs in plant cpDNAs; and (3) further investigation of molecular mechanism underlying SSC switching may reveal new driving forces for large SVs.

## Background

Chloroplast genomes (plastomes), also called chloroplast DNAs (cpDNAs) contain valuable markers for studying evolutionary relationships and population genetics of plants (1). In contrast to mitochondrial and nuclear genomes, cpDNAs across spermatophytes (*i.e.* seed plants) exhibit a higher degree of conservation with respect to their gene content, structure and organization (Asaf et al., 2017). Most cpDNAs encode about 80 protein-coding genes that are primarily involved in photosynthesis and other biochemical processes, along with 4 rRNA and 30 tRNA genes (Moghaddam et al., 2022). A plant cpDNA typically has a circular structure and includes a large single-copy region (LSC), a small single-copy region (SSC) and two inverted repeats (denoted as IR1 and IR2) separating the LSC and SSC. As the organization of these four elementary regions is highly conserved across all plant cpDNAs, LSC-IR1-SSC-IR2 is used a common structure for the assembly of chloroplast genomes. Although many structural variations (SVs) including duplications, deletions, insertions, and inversions have been reported at the levels of genome or gene between some of the angiosperm lineages, including *Asteraceae* (Ki-Joong et al., 2005), *Campanulaceae* (Haberle et al., 2008), *Onagraceae* (Stephan et al., 2008), *Fabaceae* (Cai et al., 2008) and *Geraniaceae* (Jansen, 2011), very few of the reported SVs occur at the elementary-region level, *e.g.*, a SV resulting in loss of a whole IR in a clade of *Papilionoideae* (Moghaddam et al., 2022), which is regarded as a rarely occurring event.

Cultivated rice (*Oryza sativa* L.), belonging to the grass family *Poaceae* (*Gramineae*), was domesticated from common wild rice (*Oryza rufipogon* Griff.). Dongxiang wild rice (DXWR) is a Chinese common wild rice (*O. rufipogon*) that was firstly discovered in Dongxiang county, Jiangxi province of China in 1978, which is northernmost (28°14’N) of the regions where many common wild rice population have been discovered globally. In our previous study (Zhang et al., 2016), we have compared the DXWR genome with the reference genome of cultivated rice (*O. sativa* ssp*. japonica*) and determined the loss/acquisition of genes during rice domestication by detection of SVs. However, we were not able to assembly the complete DXWR genome, as only short next-generation sequencing (NGS) data of DXWR genome was available. In order to accurately determine more genomics features of wild rice, we have initiated a project to obtain the full-length nuclear, mitochondrial and chloroplast genomes of DXWR using the PacBio DNA-seq (Xu et al., 2019). During the genome assembly, we unexpectedly discovered multiple large SVs in only a few seedlings. Accordingly, we report these very important findings for three main purposes: (1) to provide a new understanding of the conservation and variation in cpDNAs; (2) to help researchers to correctly assemble the chloroplast genomes; and (3) to initiate investigation of the molecular mechanism underlying SSC switching, leading to discover new driving forces for large SVs.

## Results

### Genome sequencing, assembly and annotation

For the *de novo* assembly of full-length nuclear, mitochondrial and chloroplast genomes of Dongxiang wild rice line 159 (DXWR159), one 500 bp and one 10 Kb DNA libraries were prepared using fresh leaves from a few (<4) seedlings of DXWR159 and sequenced on the Illumina and PacBio platforms, respectively. In the subsequent data analyses, 217,263 subreads extracted from the PacBio DNA-seq data were used to assemble the DXWR159 cpDNA with a total length of 134,509 bp at an extremely high depth of approximately 13,441 X. Then, the two IRs, long poly(GC), low complexity, and other repeat regions were exactly determined by manual curation (Methods and Materials). Long (> 20 Kb) PacBio subreads were used to validate the structure of DXWR159 cpDNA and all detected SVs. As the draft genome using high-depth PacBio data may still contain two types of errors in the low complexity and STR regions, respectively (Chang et al., 2022), 1,223,905 pairs of Illumina DNA-seq reads were properly aligned to the DXWR159 cpDNA and only one error in a STR region was corrected. The DXWR159 cpDNA is a full-length genome (**Supplementary File 1**), as defined to has no gaps and ambiguous nucleotides (Chang et al., 2022).

**Figure 1:**
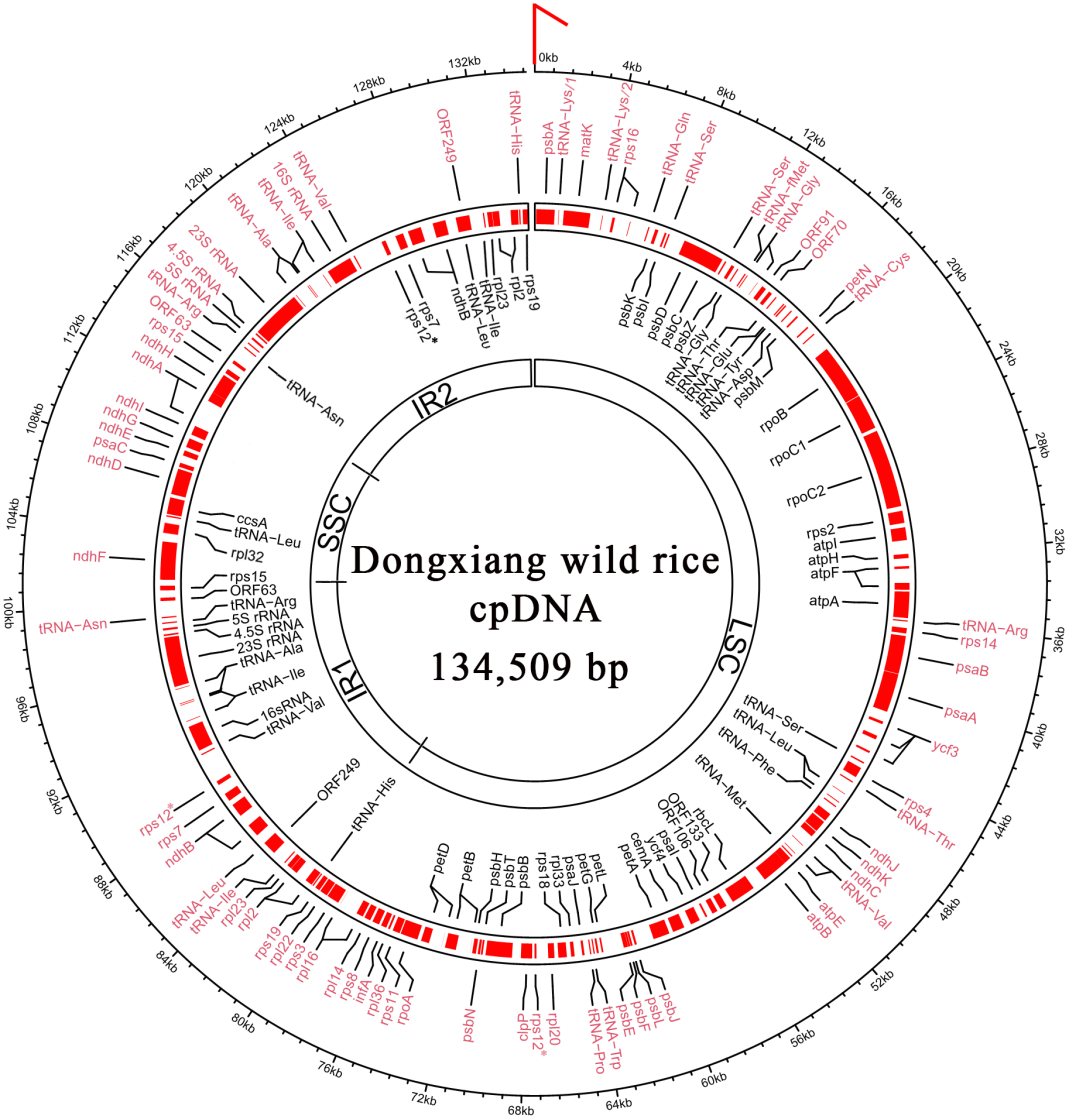
Full-length chloroplast genome of Dongxiang wild rice. According to the structure LSC-IR1-SSC-IR2, the genome sequence of circular cpDNA was clockwise linearized, starting at the first three nts “CCC” of LSC (indicated by a red arrow). In total, 4 rRNA, 30 tRNA, 76 protein-coding genes and 6 ORFs are annotated. The genes encoded on the forward strand and reverse strands are indicated by symbols written in black and red text, respectively. The genome sequences and the gene annotations are provided in the supplementary file 1 and 2, respectively. LSC, large single-copy region; SSC, small single-copy region; IR1, inverted repeat 1; IR2, inverted repeat 2. * The gene rps12 has two exons and the second exon has two copies in IR1 and IR2.

According to the NCBI RefSeq database, 4 rRNA, 30 tRNA, 75 protein-coding genes and 15 open reading frames (ORFs) have been annotated in the rice reference cpDNA (RefSeq: NC_001320) of *O. sativa* ssp*. japonica* (Nipponbare). Both DXWR159 and Nipponbare have almost identical chloroplast rRNA, tRNA and protein-coding genes (**Figure 1**). In a recent study (Moghaddam et al., 2022), 4 rRNA, 30 tRNA and 76 protein-coding genes (**Table 1**) have been well annotated in the *Onobrychis gaubae* cpDNA (GenBank: LC647182) with a length of 122,688 bp and also in the *O. viciifolia* cpDNA (GenBank: MW007721) with a length of 121,932 bp. By sequence alignment, we identified 73 of the 76 *Onobrychis* protein-coding genes as common genes which are present in both rice (DXWR159 and Nipponbare) and *Onobrychis* (*O. gaubae* and *O. viciifolia*) cpDNAs, while the three other protein-coding genes (*acc*D, *ycf*1 and *ycf*2) are absent in rice cpDNAs. Furthermore, we updated the annotations of chloroplast genes in rice and *Onobrychis* with correction, particularly: (1) the annotation of *psb*F [missed in the previous study (Moghaddam et al., 2022)] was added into the *Onobrychis* cpDNAs; (2) ORF44 in rice was identified as *psa*J; and (3) *ORF*23, *ORF*28, *ORF*56, *ORF*72, *ORF*82, *ORF*85, *ORF*100, and *ORF*137 in rice were removed.

**Table 1:** Annotated genes in cpDNAs.

Category	Group	Genes
Self-replication$	Large subunit of ribosomal proteins (8)	rpl2*, rpl14*, rpl*16*, rpl20*, rpl*23*, rpl*32*, rpl*33*, rpl*36
	Small subunit of ribosomal proteins (11)	*rps*2*, rps*3*, rps*4*, rps*7*, rps*8*, rps*11*, rps*12*, rps14*, rps*15*, rps*18*, rps*19
	DNA-dependent RNA polymerase (4)	*rpo*A*, rpo*B*, rpo*C1*, rpo*C2
	Ribosomal RNA genes (4)	rRNA 16S, 23S, 4.5S, 5S
	Transfer RNA genes (30)	30 tRNA genes (6 contain an intron)
Genes for photosynthesis$	Subunits of NADH-dehydrogenase (11)	*ndh*A*, ndhB*, ndhC*, ndh*D*, ndh*E*, ndh*F*, ndh*G*, ndh*H*, ndh*I*, ndh*J*, ndh*K
	Subunits of photosystem I (5)	*psa*A*, psa*B*, psa*C*, psa*I*, psa*J
	Subunits of photosystem II (15)	*psb*A*, psb*B*, psb*C*, psb*D*, psb*E*, psb*F*, psb*H*, psb*I*, psb*J*, psb*K*, psb*L*, psb*M*, psb*N*, psb*T*, psb*Z
	Subunits of cytochrome b/f complex (6)	*pet*A*, pet*B*, petD*, petG, *pet*L*, pet*N
	Subunits of ATP synthase (6)	*atp*A*, atp*B*, atp*E*, atp*F*, atpH*, atp*I
	Subunit of rubisco (1)	*rbc*L
Others$	Maturase K	*mat*K
	Envelope membrane protein	*cem*A
	Subunit of Acetyl-CoA-carboxylase	*acc*D
	C-type cytochrome synthesis gene	*ccs*A
	Protease	*clp P*
Unknown function$	Conserved hypothetical ORFs (4)	*ycf*1*, ycf*2*, ycf*4*, ycf*3**
Only present in Rice	Genes (3) and ORFs (6)	*inf*A*, rp*l22*, rp*s16*, ORF63, 70, 91, 106, 133, and 249

$76 protein-coding genes are present in Onobrychis cpDNAs. In the second column, the number in parentheses after the group name is the gene number of this group. In the third column, the number of asterisks after the gene names indicates the number of introns contained in the genes. The duplicated genes in two IRs are counted once. Three genes accD, ycf1 and ycf2 are absent in rice cpDNAs, while three genes infA, rpl22 and rps16 are absent in Onobrychis cpDNAs. In addition, ORF63, 70, 91, 106, 133, and 249 were predicted in rice cpDNAs. The sequences of all these genes are provided in the **Supplementary File 1**.

After correction, 4 rRNA, 30 tRNA, 76 protein-coding genes and 6 ORFs (*ORF*63, 70, 91, 106, 133, and 249) were annotated in the DXWR159 and Nipponbare cpDNAs (**Supplementary File 2**). The 76 rice protein-coding genes include 73 common genes which are present in both rice and *Onobrychis* cpDNAs, while the three other protein-coding genes (*inf*A, *rpl*22 and *rps*16) are absent in *Onobrychis* cpDNAs. Among the 76 protein-coding genes in rice, 10 multi-exon genes are *rpl*2, *rpl*16, *rps*12, *ndh*A, *ndh*B, *pet*B, *pet*D, *atp*F, and *rps*16 with two exons, and *ycf*3 with three exons (**Table 1**). The same 4 rRNA and 30 tRNA genes are present in both rice and *Onobrychis* cpDNAs. Among the 30 tRNAs, six are multi-exon genes which contain two exons, and they are tRNA^Lys^(AAA), tRNA^Gly^(GGA), tRNA^Leu^(UUA), tRNA^Val^(GUA), tRNA^Ile^(AUC), tRNA^Ala^(GCA). In addition, tRNA^Leu^ (UUA), tRNA^Leu^ (CUA), tRNA^Lys^ (AAA), tRNA^Ser^ (UCC), tRNA^Ser^ (UCA), tRNA^Ser^ (AGC), tRNA^Tyr^ (UAC) have irregular secondary structures.

### Short tandem repeats between individuals

Blasting the DXWR159 cpDNA sequence to the NCBI NT database, we found that the best hit is the cpDNA (GenBank: CP056064) of Zhenshan97, a cultivar of *O. sativa* ssp. *indica*. The length of DXWR159 cpDNA was determined to be 134,509 bp, which is very close to the Zhenshan97 and Nipponbare cpDNA lengths of 134,501 bp and 134,525 bp, respectively. The GC contents of the DXWR159, Zhenshan97, and Nipponbare cpDNAs are also very close (approximately 39%). In addition, the LSC, IR1, SSC, and IR2 of the DXWR159 cpDNA had lengths of 80,553, 20,805, 12,346, and 20,805 bp, and shared nt sequence identities of 99.93% (80505/80565), 99.99% (20804/20805), 99.99% (12346/12347), and 99.99% with those of the Zhenshan97 cpDNA, respectively. Likewise, the LSC, IR1, SSC, and IR2 of the DXWR159 cpDNA shared the nucleotide (nt) sequence identities of 99.54% (80338/80709), 99.81% (20779/20817), 99.76% (12320/12350), and 99.81% with those of the Nipponbare cpDNA, respectively. These results indicated that cpDNAs across rice species exhibit an extreme high degree of conservation with respect to their gene content, structure and organization. Multiple sequence alignment of the three cpDNAs demonstrated that about 76% (1519/2000) of the variation sites were single nucleotide polymorphisms (SNPs), while the others were small insertions and deletions (InDels). Almost all the InDels were associated with copy number variation (CNV) of STRs. STRs (Chen et al., 2020), which are widely used by forensic geneticists and genealogy experts, are often referred to as simple sequence repeats (SSRs) by plant geneticists or microsatellites by oncologists. The minimum repeat unit length of STR is evidently 1 bp; this type of STR is predominantly referred to as a polynucleotide (*e.g.* polyAs and polyTs).

Subsequently, we detected the variation sites across two lines of DXWR, DXWR159 and DXWR line 44 (DXWR44). Aligning the NGS data of DXWR44 (Materials and Methods) to the DXWR159 cpDNA, we found that DXWR159 and DXWR44 share an identical chloroplast genome without copy number variation (CNV) of STRs or SNPs between them. We then detected CNV of STRs and SNPs in cpDNAs of DXWR159 and DXWR44, respectively. Most of the detected STRs with CNV were polyAs or polyTs, but SNPs were not detected. Among these polyAs and polyTs, at least nine were shared by DXWR159 and DXWR44. They were 11291[T]_7-8_, 31462[T]_7-8_, 36488[T]_9-10_, 49216[T]_10-15_, 63462[A]_6-9_, 80673[A]_2-3_, 102303[A]_6-8_, 107081[A]_6-7_ and 111165[A]_6-8_, where a STR (*e.g.*, 11291[T]_7-8_) is described by its genomic position in numbers (*e.g.*, 11291 in 11291[T]_7-8_), the repeat unit [in brackets] (*e.g.*, [T] in 11291[T]_7-8_), and its copy numbers as subscripts (*e.g.*, 7-8 in 11291[T]_7-8_), as described in our previous study (Chen et al., 2020). We inferred that CNV of STRs, particularly polyAs or polyTs occurs more frequently than we expected, and accounts for cpDNA diversity within an individual of plants, just as it does to mtDNA diversity within an individual of tick, insect and human, which has been reported in our previous study (Chen et al., 2020).

### Large structural variations and SSC switching

**Figure 2:**
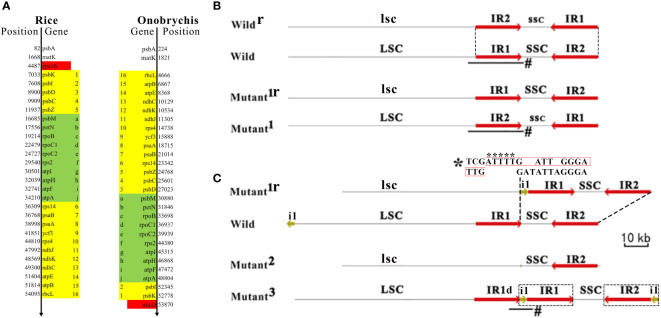
Large structural variations in cpDNAs and their formation. Plant chloroplast DNA (cpDNA) typically has a circular structure, including a large single-copy region (LSC), a small single-copy region (SSC) and two inverted repeats (IR1 and IR2). The organization of these four elementary regions LSC-IR1-SSC-IR2 is highly conserved across all plant cpDNAs. **(A)** A large inversion resulted in a reverse orientation of 26 genes (from *psbK* to *rbcL*) in the LSCs and the loss/acquisition of *rps*16 and *accD* (in red color). Another large inversion resulted in a reverse orientation of 10 genes (in green color). The genomic positions of the genes were annotated according to the Nipponbare cpDNA (RefSeq: NC_001320) and the *Onobrychis gaubae* cpDNA (GenBank: LC647182). **(B)** A large inversion of SSC results in a reverse orientation of SSC (denoted as ssc). According to the orientation of SSC, cpDNAs can be classified into the forward SSC (SSC-F) and reverse SSC (SSC-R) type cpDNAs, which were also designated as wild-type cpDNAs (denoted as wild) and mutants (denoted as mutant^1^). The frequent inversion of a whole SSC was named as SSC switching. Wild^r^ and mutant^1r^ represents cpDNAs that have reverse complimentary sequences to wild and mutant^1^, respectively. LSC and SSC are reverse complimentary to lsc and ssc, respectively. As IR1 is identical to IR2, both LSC-IR1-ssc-IR2 and LSC-IR2-ssc-IR1 represent the same sequence **(C)** The recombination between mutant^1r^ and wild resulted in a cpDNA (mutant^2^) that lost a IR and another (mutant^3^) that acquired a IR. Although the IR−lacking cpDNA (mutant^2^) was not detected in the present study, it had been previously reported in *Onobrychis* spp (Moghaddam et al., 2022). While the 319-bp deleted segment was at the 3’ end of the IR1 the 22810-bp insert includes five nucleotides “ATTTT” plus a 2000-bp segment of LSC (denoted as i1) and a whole IR1. The 319-bp lacking IR1 is denoted as IR1d. A high-score segment pair (HSP) that may be involved as recombination sites (indicated by *) was detected between the 5’ end of the 319-bp deleted segment (the below sequence) and that of i1 (the above sequence). Junction reads (indicated by #) were used to validate these structural variations (Materials and methods). The sequence (in black box) synthesized during recombination contain an insert of five nucleotides “ATTTT” (indicated by asterisks below) that resulted in a repeat TTGAATTTTGATT.

The comparative genomics analysis revealed many large (> 10 Kb) SVs between rice and *Onobrychis* cpDNAs. Among these SVs, three large inversions (named as SV1, SV2 and SV3) were notable in size: SV1 resulted in a reverse orientation of 26 genes in the LSCs and the loss/acquisition of two genes (*rps*16 and *accD*) (**Figure 2A**); SV2 resulted in a reverse orientation of 10 genes (**Figure 2A**); and SV3 resulted in a reverse orientation of *Onobrychis* SSC, compared to the rice SSC (**Supplementary File 2**). The other SVs were much smaller than the three SVs in size. Among these smaller SVs, two deletions resulted in the loss of *ycf*1 and *ycf*2. The two genes (*ycf*1 and *ycf*2) are absent in rice cpDNAs, while *ycf*3 and *ycf*4 are present in both rice and *Onobrychis* cpDNAs. The genes *ycf*1-4 are the most common members of the hypothetical chloroplast reading frames. Both Nipponbare and *Onobrychis gaubae* have *ycf*3 with a comparatively high nt sequence identity of 89% (447/505), and *ycf*4 with a very low identity (far less than 70%). As the only protein-coding gene containing three exons in these cpDNAs (**Table 1**), *ycf*3 is highly conserved in its nt sequences across kingdoms, indicating that *ycf*3 may has important biological functions.

Subsequently, two important large SVs (named as SV4 and SV5) were discovered in DXWR159 cpDNAs. SV4 was a large inversion of SSC, resulting in a reverse orientation of SSC. According to the orientation of SSC, cpDNAs can be classified into the forward SSC (SSC-F) and reverse SSC (SSC-R) type cpDNAs, which were also designated as wild-type cpDNAs and mutants (**Figure 2B**), respectively. The organization of Nipponbare cpDNA was defined as the SSC-F type structure (denoted as LSC-IR1-SSC-IR2). Accordingly, the organization of a cpDNA with a SSC reverse to that of the Nipponbare cpDNA was defined as the SSC-R type structure (denoted as LSC-IR1-ssc-IR2). According to the designations, the two *Onobrychis* cpDNAs (Described above) have the SSC-R structures. As the most significant finding, both SSC-F and SSC-R type cpDNAs were detected in only several seedlings of DXWR159. Then, we used long (> 20 Kb) PacBio subreads as junction reads spanning the LSC-IR1-SSC and LSC-IR1-ssc regions (**Figure 2B**) for validation (Materials and methods). As a result, these junction reads (**Supplementary File 3**) demonstrated the presence of both SSC-F (n = 82) and SSC-R type (n = 99) cpDNAs in similar proportions of approximately 45% to 55%. The SSC/ssc ratio of 0.82 (0.45/0.55) suggested that the inversion of a whole SSC is not a rarely occurring event and may occur frequently. We used SSC switching to name the frequent inversion of a whole SSC, as it is analogous to the mating-type (MAT) region in a yeast genome (Haber, 2012). MAT switching and SSC switching occur in similar structures IR-MAT-IR and IR-SSC-IR, respectively. The MAT region has a length of ~19 Kb between two IRs, while the SSC has a length of ~12.4 Kb between two IRs. During MAT or SSC switching (**Figure 2B**), the two IRs recombine, inverting MAT or SSC relative to the rests of the chromosomes. Neither MAT nor SSC switching changes the sequences of genes, as the structure of IR-MAT-IR or IR-SSC-IR is symmetric. By MAT switching, yeasts initiate the transcription of some mating related genes, determining the sexual identity of a cell. The molecular mechanism underlying MAT switching has already been revealed by scientists (Haber, 2012). However, the molecular mechanism underlying SSC switching is unknown.

Further analysis showed that rice cpDNAs are highly conserved on the junction site between IR1 and SSC (IR1-SSC) or that between SSC and IR2 (SSC-IR2). As rice cpDNAs are linearized for their representation, starting at the first three nts “CCC” of LSC, almost all the IR1-SSC junction sites contain the highly conserved sequences of TGGAAAAAATCG|GCAAATAGGAAA and TGGAAAAAATCG|CGGAAAACCGAA for the SSC-F and SSC-R types, respectively. This feature was then used to quickly screen rice cpDNAs from the NCBI GenBank database. The results revealed that the structures of most rice cpDNAs belong to the SSC-F type, whereas very few (e.g. Zhenshan97) belong to the SSC-R type. The notable SSC-F type cpDNAs are the cpDNAs of *O. sativa L.* spp. *indica* (GenBank: JN861110), *O. nivara* (KF359901), *O. glaberrima* (KF359903), *O. barthii* (KF359904), *O. glumaepatula* (KF359905), *O. meridionalis* (KF359906), *O. punctata* (MT726932) and *O. brachyantha* (MT726939). However, The SSCs in some of the SSC-F type cpDNAs are chimeras. For instance, *O. nivara* and *O. punctata* have IR1-SSC junction sites of the SSC-R type cpDNAs, but SSC bodies of the SSC-F type cpDNAs (**Supplementary file 1**). This suggested that these SSCs had been assembled using hybrid data from both the SSC-F and SSC-R type cpDNAs. Based on the above results, we proposed that: (1) SSC switching ubiquitous occurs in plant cpDNAs, resulting in the presence of both SSC-F and SSC-R type cpDNAs in different proportions; and (2) an assembled chloroplast genome only represents the dominant type of cpDNAs. However, most of cpDNAs have been “*de novo*” assembled using short NGS or Sanger sequencing data and the orientations of SSCs have been determined according to the structure LSC-IR1-SSC-IR2 without validation using junction reads. According to the classical definition, *de novo* assembly refers to the process of reconstructing a genome without knowing its structure. For instance, the Zhenshan97 cpDNA (GenBank: CP056064) was *de novo* assembled using PacBio DNA-seq data. We inferred that the SSC-F type cpDNAs of Zhenshan97 are supposed to be present as the SSC-R type cpDNAs, in a comparatively smaller proportion.

SV5 was a large duplication of IR in cpDNAs of several DXWR159 seedlings (**Figure 2C**). Compared to the wild-type cpDNA, the mutant acquired a 319-bp deletion (denoted as d1) and a 22810-bp insertion. While the 319-bp segment was deleted from the 3’ end of the IR1 (**Figure 2C**), the 22810-bp insert included five nucleotides “ATTTT” plus a 2000-bp segment of LSC (denoted as i1) and a whole IR1. Six long (> 20 Kb) PacBio subreads (**Supplementary File 1**) were used as junction reads for validation (Materials and Methods). As a result, these junction reads spanned the IR1d-i1-IR1 region (**Figure 2C**), validating this new finding. Using the blast program, a high-score segment pair (HSP) that may be involved as recombination sites (**Figure 2C**) was detected between the 5’ end of the 319-bp deleted segment and that of i1. This inspired us to propose a homologous recombination model to explain the formation of SV4 and SV5. This model includes symmetric recombination and asymmetric recombination. Symmetric recombination is reversible, while asymmetric recombination is irreversible. Symmetric recombination occurs frequently in symmetric regions (*e.g.* IR1-SSC-IR2 and IR1-ssc-IR2) of cpDNAs (**Figure 2B**), repeatedly resulting in the SSC-F and SSC-R structures (*i.e.*, SSC switching). Therefore, both structures are ubiquitous present in plant cpDNAs. However, recombination rarely occurs in asymmetric regions (*e.g.* i1-IR1-SSC-IR2 and d1-SSC-IR2), which was defined as asymmetric recombination (**Figure 2C**). Asymmetric recombination results in loss/acquisition of large segments of cpDNAs. According to this model, an asymmetric recombination between two cpDNAs resulted in an exchange between i1+IR1 and d1. Consequently, one of the two cpDNAs acquired a large duplication of IR, whereas the other lost an IR, presumably resulting in an IR−lacking cpDNA. Although IR−lacking cpDNAs were not detected in the present study, an IR−lacking cpDNA had been previously reported in *Onobrychis* spp (Moghaddam et al., 2022), confirming the prediction of our model. Our recombination model explained the formation of large SVs in cpDNAs and revealed the association between symmetric recombination and asymmetric recombination.

One of two additional findings is that the i1-IR1 region is the invert repeat of the IR2-i1 region, indicating that the second large SV resulted in IR expansion. Asymmetric recombination maybe a cause of IR expansion. The other additional finding is that the sequence synthesized during recombination contain an insert of five nucleotides “ATTTT” that resulted in a tandem repeat TTGATTTTGATT (**Figure 2C**), which merits further investigation. Further analysis revealed that the tandem repeat may has a breakpoint “TGG|ATT” for enzyme cleavage at 5’ end of the IR1. Both SSC switching and MAT switching occur in featured DNA regions, which were defined as DNA transposon-like elements (TLEs), as they have similar structures as DNA transposon elements (TEs). We defined that a TLE is composed of an internal coding region bounded by two flanking IRs but does not include transposase genes in the coding region. As both IR-SSC-IR and IR-MAT-IR are TLEs, we addressed a novel scientific question that if there are common mechanisms in the formation or translocation of all kinds of TLEs. To answer this question, the first step is to validate our hypothesis that symmetric recombination of TLEs results in TLE switching, while asymmetric recombination of TLEs results in loss/acquisition of large segments and possible IR expansion. The validation requires intensive research of a variety of TLEs in nuclear, chloroplast and mitochondrial genomes. One of our ongoing studies is to detect the switching of a transposon-like element 1 (TLE1) in tick mitochondrial genomes using PacBio DNA-seq. TLE1, as the first reported TLE in mitochondrial genomes (Chen et al., 2020), includes genes ND1, tRNA^Leu^, 16S rRNA, tRNA^Val^, 12S rRNA, CR1, tRNA^Ile^, tRNA^Gln^ in the coding region with two flanking IRs. The IRs contains the breakpoint-like sequenc “TGCA|” at their 5’ ends. Based on the above results, we proposed that SSC switching, MAT switching or even more TLEs may share common mechanisms. The most possible mechanism is synthesis-dependent strand-annealing (SDSA), which has been well studied in MAT switching in previous studies (Haber, 2012). SDSA in yeasts initiates when HO endonuclease makes a double-stranded DNA break at the MAT locus (Haber, 2012). As DNA synthesis in SDSA does not use all the factors employed in normal replication, the possible errors in synthesized DNA can not be corrected or removed. The insert of five nucleotides “ATTTT” may be a mutation caused by errors in synthesized DNA by SDSA. Another possible mechanism may depend on the transposase system, which has not been reported in the formation or translocation of any TLE. However, as recombinases, transposases and other enzymes with similar functions contain common structural domains (e.g. homeodomain), the complexity of the system goes beyond what is known based on existing knowledge. Thus, further studies of symmetric and asymmetric recombination may provide a new research direction to unravel the underlying mechanisms.

## Conclusions

In the present study, we assembled the full-length chloroplast genome of Dongxiang wild rice, a Chinese common wild rice. The two main findings included the CNV of STRs between individuals of DXWR159 and two important large SVs at the elementary-region level. Large SVs and CNV of STRs, particularly polyAs or polyTs occur more frequently than we expected within an individual of plants, which merits further investigation in future studies. As CNV of STRs occurs frequently in an individual, STRs in cpDNAs may not be useful as molecular markers in phylogenetic studies, particularly at low taxonomic levels (*e.g.*, between individuals, lines or species). As the most significant finding, both SSC-R and SSC-F type cpDNAs were detected in similar proportions in several DXWR159 seedlings. The frequent inversion of a whole SSC was named as SSC switching. SSC switching occurs much more frequently than we expected within an individual of plants.

We proposed a homologous recombination model to explain the formation of two important large SVs. This model includes symmetric recombination and asymmetric recombination. Symmetric recombination is reversible, while asymmetric recombination is irreversible. Symmetric recombination results in SSC switching, while asymmetric recombination results in loss/acquisition of large segments of cpDNAs and possible IR expansion. The SSC/ssc ratio may indicate the activities of the enzymes that are responsible for SSC switching and asymmetric recombination. Our recombination model explained the formation of large SVs in cpDNAs and provided insights into a novel scientific question that if there are common mechanisms in the formation or translocation of all kinds of TLEs. We propose that: (1) large inversion is the most accepted mutation type of SVs in cpDNAs; (2) SSC switching ubiquitous occurs in plant cpDNAs; and (3) further investigation of molecular mechanism underlying SSC switching may reveal new driving forces for large SVs. As most species may have both SSC-F and SSC-R type cpDNAs, we recommend researchers to assemble the reference cpDNA using the SSC-F structure of the species rather than using the dominant structure of their samples. In addition, researchers can report the proportions of SSC-F and SSC-R type cpDNAs by counting the junction reads (Materials and Methods).

## Materials and methods

All specimen used in the present study was identified by Fantao Zhang. DXWR159 and DXWR44 are two rice lines isolated from the Dongtangshang and Anjiashan populations of Dongxiang wild rice, respectively. DNA extraction and quality control were performed as described in our previous study (Wang et al., 2016). A 500 bp DNA library of DXWR44 was constructed as described in our previous study (Zhang et al., 2016) and sequenced on the Illumina HiSeq 2000 platform to produce 90-bp paired-end data. A 350 bp DNA library of DXWR159 was constructed and sequenced on the Illumina HiSeq X Ten platform to produce 150-bp paired-end data. A 10 Kb DNA library of DXWR159 using fresh leaves from a few (<4) seedlings was constructed and sequenced on the PacBio Sequel platform, according to the manufacturer’s instructions. The cleaning and quality control of PacBio DNA-seq data were performed with the software SMRTlink v5.0, while the cleaning and quality control of Illumina data was performed with the software Fastq_clean (Zhang et al., 2014) v2.0.

The PacBio DNA-seq data of DXWR159 was used to assemble the chloroplast genome with the software CANU (Koren et al., 2017) v2.2. The alignment of Illumina and PacBio DNA-seq data were performed with the software BWA (Li and Durbin, 2010) v0.7.10. Perl scripts for processing PacBio data in a special folder were integrated into Fastq_clean v2.0. Genome graphs (*i.e.*, **Figure 1**) were plotted using the software Circos (Krzywinski et al., 2009) v0.66. Statistics and plotting were conducted using the software R v2.15.3 with Bioconductor packages (Gao et al., 2014). Using the software Tablet (Milne et al., 2013) v1.17, manual curation of the two IRs, long poly(GC), low complexity, and other repeat regions were performed. Long (> 20 Kb) PacBio subreads were used as junction reads to validate the structure of DXWR159 cpDNA and all detected SVs. To validate SVs (*e.g.* LSC-IR1-SSC and LSC-IR1-ssc), junction reads are required to be aligned to the SSC-F and SSC-R cpDNAs of DXWR159 with more than 90% of their lengths and cover 100% of IR1 with two 200-bp long overhangs that can be aligned to LSC and SSC/ssc at the 5′ and 3′ ends of IR1, respectively (**Figure 2B**). These aligned junctions reads were provided in SAM format with the SSC-F and SSC-R cpDNAs of DXWR159 (**Supplementary file 3**), which can be observed using the software Tablet.

## Data availability statement

The datasets presented in this study can be found in online repositories. The NGS data are available in the NCBI SRA database with ID SRP070627. All the supporting data are included as additional files.

## Ethics statement

All research on the rice lines detailed in this manuscript comply with the IUCN Policy Statement on Research Involving Species at Risk of Extinction and the Convention on the Trade in Endangered Species of Wild Fauna and Flora. All lines of Dongxiang wild rice (DXWR) are preserved *ex situ* at Jiangxi Academy of Agricultural Sciences, Nanchang, China (http://www.jxaas.com/index.html), and the seeds of DXWR are freely available for scientific research.

## Author contributions

SG conceived this project. SG and SY supervised this study. JL, QW, and YY analyzed the data. RC performed the programming. FZ conducted experiments. SG drafted the main manuscript. SG revised the manuscript. ML prepared all the figures, tables and additional files. All authors have read and approved the manuscript.

## Funding

This work was supported by the Natural Science Foundation of Jiangxi Province, China (20202ACB205002) to Fantao Zhang and the Innovation Research and Experiment Program for Youth Scholar, Tianjin Academy of Agricultural Sciences (2021023) to Qian Wang. The funding bodies played no role in the study design, data collection, analysis, interpretation or manuscript writing.

## Acknowledgments

We appreciate the help from Professor Jiankun Xie from Jiangxi Normal University and Professor Wenjun Bu in College of Life Sciences, Nankai University. This manuscript was online as a preprint on March 20^nd^, 2022 at Research Square with the DOI 10.21203/rs.3.rs-1470820/v1.

## Conflict of interest

The authors declare that the research was conducted in the absence of any commercial or financial relationships that could be construed as a potential conflict of interest.

## Publisher’s note

All claims expressed in this article are solely those of the authors and do not necessarily represent those of their affiliated organizations, or those of the publisher, the editors and the reviewers. Any product that may be evaluated in this article, or claim that may be made by its manufacturer, is not guaranteed or endorsed by the publisher.

## Supplementary material

Supplementary Data Sheet S1 Contains all the sequences to support the results.

Supplementary Data Sheet S2 Contains the corrected annotations of the DXWR159 and Nipponbare cpDNAs.

Supplementary Data Sheet S3 Contains all junction reads (in the ssc-f.sam and ssc-r.sam files) aligned to the SSCF and SSC-R cpDNAs (in the ssc-f.fa and ssc-r.fa files) of DXWR159 to validate LSC-IR1-SSC and LSC-IR1-ssc.

## Abbreviations

DXWR, Dongxiang wild rice; cpDNA, chloroplast DNA; LSC, large single-copy region; SSC, small single-copy region; IR1, inverted repeat 1; IR2, inverted repeat 2; SSC-F, forward SSC; SSC-R, reverse SSC; InDel, small insertion and deletion; SNP, nucleotide polymorphism; STR, short tandem repeat; SSR, simple sequence repeat; CNV, copy number variation; SV, structural variation; ORF, open reading frame; NGS, next-generation sequencing.
